# Inhibition of infection-associated oral bacteria adhesion by probiotics: *In vitro* and *in vivo* models

**DOI:** 10.1016/j.isci.2025.112412

**Published:** 2025-04-11

**Authors:** Valeriia Zymovets, Olena Rakhimova, Alexej Schmidt, Vicky Bronnec, Nataliia Limanska, Malin Brundin, Peyman Kelk, Maréne Landström, Nelly Romani Vestman

**Affiliations:** 1 Department of Odontology, Umeå University, Umeå, Sweden; 2 Department of Medical Biosciences, Pathology, Umeå University, Umeå, Sweden; 3 Department of Microbiology, Virology and Biotechnology, Odesa National I.I. Mechnykov University, Odesa, Ukraine; 4 Department of Medical and Translational Biology, Umeå University, Umeå, Sweden; 5 Wallenberg Centre for Molecular Medicine, Umeå University, Umeå, Sweden

**Keywords:** Oral microbiology, Stem cells research

## Abstract

Oral health in immature permanent teeth with traumatic injuries is particularly vulnerable, and regenerative endodontic treatment (RET) using stem cells from the apical papilla (SCAP) holds potential for root development and tissue regeneration. However, bacterial persistence, especially *Enterococcus faecalis*, poses a challenge to successful treatment outcomes. To address this, we evaluated the probiotic *Lactobacillus gasseri* for its co-aggregative and anti-adhesive properties against *E. faecalis*. An *in vitro* aggregation test demonstrated effective co-aggregation between the probiotic and opportunistic strains. Additionally, flow cytometry analysis revealed that *E. faecalis* binding to SCAP was significantly reduced when the *L. gasseri* concentration was nine times higher. To substantiate these findings, an *in vivo Drosophila melanogaster* gut model was used, where immunofluorescence imaging and culture-based methods confirmed decreased *E. faecalis* adhesion at both 1:1 and 9:1 probiotic-to-opportunistic ratios. These results highlight *L. gasseri* B16 as a promising probiotic strain to improve RET outcomes.

## Highlights

• *L. gasseri* exhibits co-aggregative properties toward oral opportunistic bacteria
• *L. gasseri* reduces the binding ability of *E. faecalis* to SCAP *in vitro*
• *E. faecalis* binding is decreased by *L. gasseri* influence based on the *in vivo* model
• N-acetylcysteine significantly reduces aggregation between *E. faecalis* and *L. gasseri*

## Graphical abstract

**Figure:**
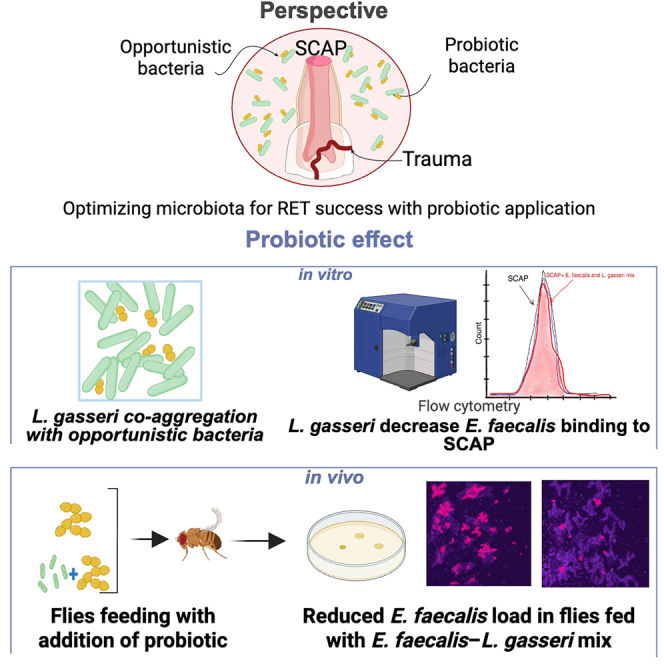

## Introduction

Due to its unique combination of hard and soft tissues, the oral cavity provides a complex niche for a diverse array of microorganisms, including bacteria, fungi, archaea, and viruses.^1^ More than 700 microorganisms comprise an elaborate net of interactions, which normally leads to sustained homeostasis and mutual benefits for microorganisms and the host.^2^^,^^3^ However, the imbalance between commensal and opportunistic bacteria leads to the occurrence of dysbiosis which eventually can result in a variety of diseases such as halitosis (bad breath), dental caries, oral ulcers, and periodontitis.^4^ Furthermore, the state of the oral microbiota holds particular significance, especially in cases involving traumatic dental injuries (TDI), as treatment outcomes may rely heavily on the oral microbial environment. In the case of TDI, oral microorganisms may reach injured pulp tissue and root canals via dentinal tubules, lateral and accessory canals, injured periodontal tissue, and even tooth cracks.^5^^,^^6^

In case of pulp necrosis occurring in young and immature teeth, two approaches have been proposed: either apexification with the use of mineral trioxide aggregate (MTA) or regenerative endodontic treatment (RET).^7^^,^^8^^,^^9^ The latter aims to stimulate the regeneration of the pulp complex through a combination of stem cells, biomimetic scaffolds, and growth factors.^10^ Regardless of the chosen method, ensuring the reducing number of bacteria is the critical step in preventing treatment failure.^5^ Currently, to prevent bacterial infection in TDI, both the systematic use of antibiotics^11^^,^^12^^,^^13^ and local intracanal antimicrobial substances are used.^14^^,^^15^^,^^16^ However, despite the broad effectiveness of standard antimicrobial agents against anaerobic bacteria in root canals,^17^ these antimicrobial treatments can have cytotoxic activity on stem cells from the dental apical papilla (SCAP), a crucial aspect in successful a RET outcome.^18^^,^^19^^,^^20^ Moreover, most microorganisms isolated from infected root-filled teeth show resistance to common antibiotics, serving as potential antibiotic resistance reservoirs.^21^

Considering the crucial role of preserving the viability and differentiation potential of SCAPs, as well as the necessity of effective bacterial reduction, it is imperative to explore new treatment strategies to improve RET outcome. One of the alternative approaches for combating opportunistic bacteria in the oral cavity can be the use of probiotic bacteria, which are beneficial microorganisms intended to maintain a healthy microbial balance in the mouth.^22^

Probiotics restore oral microbiota balance and combat oral diseases through various strategies, including the production of bacteriocins and acids, as well as competing with pathogenic bacteria for adhesion sites in the oral cavity, thereby preventing their binding.^23^^,^^24^^,^^25^ Bacteria of genus *Bifidobacterium* and *Lactobacillus* generally-recognized-*as*-safe (GRAS) and non-pathogenic^26^ are commonly used as probiotics.^27^ *Lactobacilli* are Gram-positive, rod-shaped, non-spore–forming and aerotolerant anaerobes,^28^ that mostly colonize the gastrointestinal tract and female genital tract.^29^ Commonly used probiotic *Lactobacillus* species in oral healthcare include *Limosilactobacillus reuteri*, *Lactobacillus acidophilus*, *Lacticaseibacillus paracasei*, and others.^30^ It is important to mention that it has not yet been definitively established whether probiotic species of bacteria are normal representatives of the oral microbiota. This could be due to the fact that *Lactobacilli* presence in the oral cavity has been associated with an increased risk of caries development due to the production of acids by these bacteria.^31^^,^^32^^,^^33^^,^^34^

With the existing schemes for disinfection in RET, it has been shown that it is impossible to completely eliminate the bacteria that reside in immature permanent teeth.^35^ Bacterial genera of *Fusobacterium*, *Streptococcus*, *Dialister, Filifactor*, *Prevotella*, *Parvimonas*, *Propionibacterium*, and *Pyramidobacter* were detected in teeth with primary endodontic infections,^36^ while *Enterococcus faecalis* appeared to be the most frequently identifiable bacteria from secondary infections.^37^ *E. faecalis* is a gram-positive coccus, facultative anaerobe, originally found in the intestine, where it is considered a subdominant bacterial species.^38^^,^^39^ In this regard, *E. faecalis* is a transient component of the oral microbiome and has been implicated in oral diseases as one reason for caries, periodontitis, peri-implantitis, and endodontic infections^40^^,^^41^^,^^42^ and has been frequently implicated in root canal treatment failure.^43^ *E. faecalis* demonstrates the ability to withstand adverse conditions^44^^,^^45^ and exhibits resistance to common antimicrobial substances utilized in endodontic treatment.^46^^,^^47^ Moreover, this bacterium has the ability to occupy dentinal tubules and form biofilms, posing a significant challenge in reducing bacterial numbers during endodontic treatment.

To counteract *E. faecalis* and other pathogenic bacteria, it is worthwhile to explore a novel approach involving the aggregation of pathogenic bacteria with probiotics. Aggregation is one of the mechanisms of probiotic bacteria through which they can prevent adhesion of pathogens to host tissues.^48^ In this regard, previous studies have demonstrated the probiotic’s ability to co-aggregate with oral bacteria^49^*,* but also to decrease bacterial load of oral bacteria.^50^^,^^51^ In our work, we utilized probiotic species *L. gasseri*, that has been shown to exert probiotic traits in attaching to epithelial cells, pathogen growth and most importantly – pathogen adhesion inhibition.^52^^,^^53^^,^^54^ Previous studies have shown that *L. gasseri* was able to inhibit the growth of *Fusobacterium nucleatum*,^55^ but also to control oral inflammation and bone resorption, mediated by *Porphyromonas gingivalis*.^56^ Additionally, *L. gasseri* has shown its activity in preventing *P. gingivalis*-associated periodontal disease.^56^ These findings suggest that *L. gasseri* is a promising candidate for use as a probiotic to improve oral health.

Derived from the co-aggregation phenomenon, the aim of the study was to evaluate the co-aggregative and anti-adhesive abilities of clinically isolated probiotic strain—*L. gasseri*—toward opportunistic oral isolate of *E. faecalis* using *in vitro* and *in vivo* models. Testing the efficacy of probiotic strains against crucial bacteria in RET failure will allow us to investigate the role of probiotics as key players in bacteriotherapy of oral health to increase the effectiveness of RET.

## Results

### Probiotic *L. gasseri* exhibit co-aggregative properties toward *E. faecalis*

To evaluate the ability of probiotic bacteria to influence co-aggregation with oral opportunistic species *in vitro*, we performed a qualitative bacterial aggregation assay. The assay was conducted by testing auto-aggregation properties of *L. gasseri* and *E. faecalis*, as well as the combination of *L. gasseri* with *E. faecalis* to assess their co-aggregation potential.

**Figure 1:**
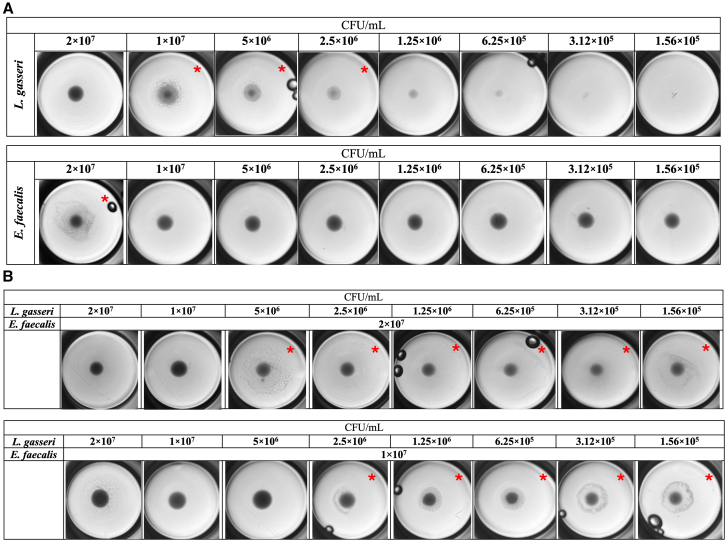
Aggregation test of probiotic and opportunistic bacteria at various concentrations and combinations (A) Auto-aggregation test of *L. gasseri* and *E. faecalis* mono-suspensions. Each well represents a serial 2-fold dilution of the bacterial suspensions, starting from the highest concentration to the lowest (left to right). (B) Co-aggregation test of *L. gasseri*, gradually diluted 2-fold, mixed with *E. faecalis* suspensions at concentrations of 2 × 10^7^ CFU/mL and 1 × 10^7^ CFU/mL. Wells showing visible bacterial aggregation are marked with a red star.

Using the aggregation test criteria, which assess the formation of a thin bacterial layer at the bottom of the well, it was demonstrated that *L. gasseri* exhibited partial auto-aggregative properties up to a dilution of 2.5 × 10^6^ CFU/mL, while visible auto-aggregation of *E. faecalis*, although partial, was only observed at the highest concentration of 2 × 10^7^ CFU/mL (Figure 1A).

We subsequently assessed the co-aggregative properties of *L. gasseri* in gradual dilutions with different concentrations of *E. faecalis*. The aggregation assay demonstrated that partial co-aggregation occurred when *E. faecalis* at a concentration of 2 × 10^7^ CFU/mL was mixed with *L. gasseri* dilutions ranging from 5 × 10^6^ CFU/mL to 1.56 × 10^5^ CFU/mL. Furthermore, *E. faecalis* at a concentration of 1 × 10^7^ CFU/mL showed more evident partial co-aggregation with *L. gasseri* dilutions ranging from 2.5 × 10^6^ CFU/mL to 1.56 × 10^5^ CFU/mL (Figure 1B). Different dilutions of *L. gasseri* tested with *E. faecalis* concentrations below 1 × 10^7^ CFU/mL, did not exhibit any co-aggregation features (Figure S1).

### Probiotic bacteria *L. gasseri* influence the ability of *E. faecalis* binding to SCAP

**Figure 2:**
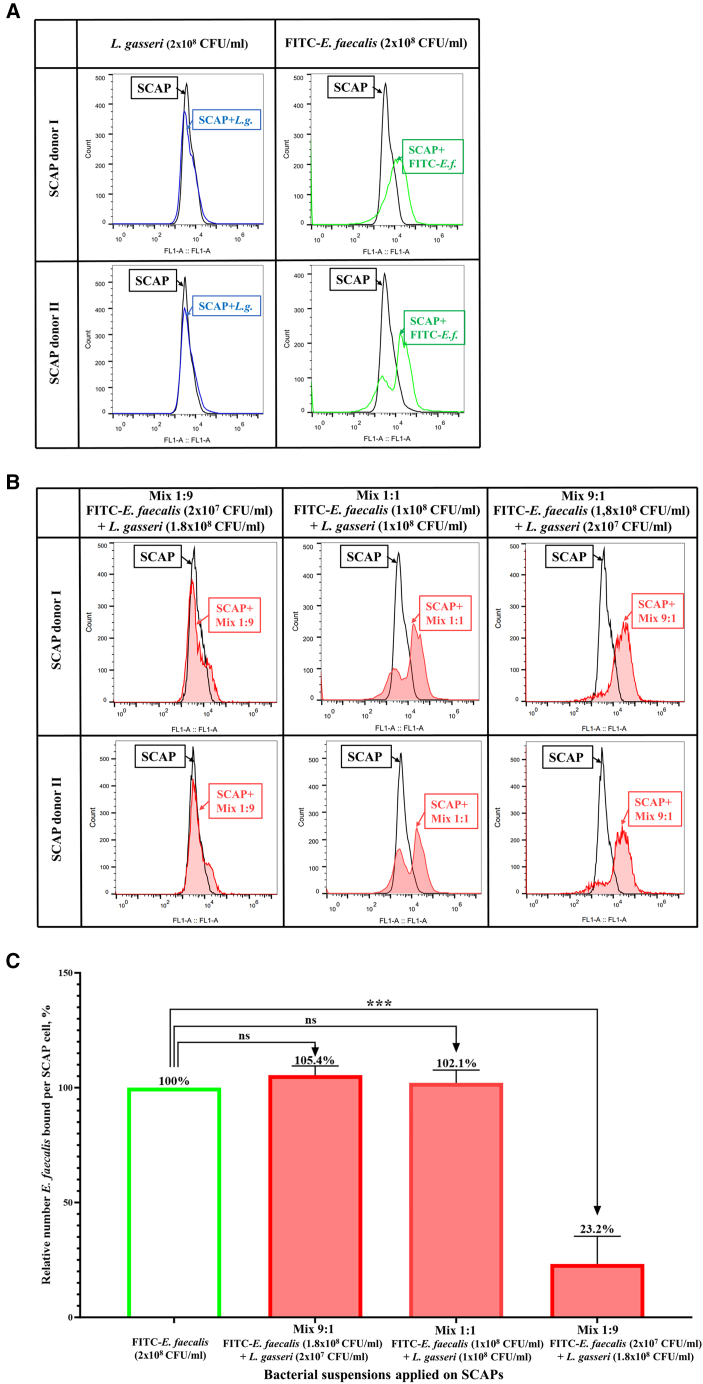
Binding of FITC-labeled *E. faecalis* to SCAPs, either as a mono suspension or in combination with *L. gasseri* at different proportions; SCAP without bacteria marked by the black line (A) Flow cytometry histograms of controls – SCAPs treated by bacterial mixtures of *L. gasseri* (green line) or *E. faecalis* (blue line). (B) Flow cytometry histograms of SCAPs treated with different mixtures of FITC-labeled *E. faecalis* and *L. gasseri* (1:9, 1:1 and 1:9)—by the red line with pink area chart. (C) Numbers of FITC-labeled *E. faecalis* per SCAP cell were calculated for each treatment variant. The results are presented as the mean values ±SD. Dunnett’s multiple comparisons test was used for statistical analysis. ∗∗∗ (*p* < 0.001) indicates a significant difference, while ‘ns’ denotes a non-significant result.

Based on the co-aggregation assay results, we performed flow cytometry (FC) assay in order to analyze if co-aggregation can decrease the binding of *E. faecalis* to SCAP in the presence of *L. gasseri*. First, we investigated whether non-labeled *L. gasseri* could bind to SCAP and potentially interfere with the fluorescent signal emitted by SCAPs, causing a noticeable shift. Our tests confirmed that such a shift did not occur (Figure 2A, blue line on the histograms). Subsequently, we observed that treatment of SCAPs with a mono-suspension of FITC-labeled *E. faecalis* led to a shift in fluorescent intensity signal to the right, indicating the binding of *E. faecalis* to SCAPs (Figure 2A, green line on the histograms). However, when treated with a 1:9 mixture, there was no extreme shift in fluorescent intensity compared to the *L. gasseri* control (Figure 2B, red line on the histogram).

By calculating the relative number of bound *E. faecalis*, we found that treatment of SCAPs with a 9:1 mixture did not result in a decrease in bound *E. faecalis* (Figure 2C). Interestingly, when SCAPs were treated with a 1:1 mixture, where the concentration of *E. faecalis* was two times lower than in the 9:1 mixture, we did not observe a corresponding decrease in bound *E. faecalis* (Figure 2C). Only when SCAPs were exposed to a mixture of FITC-labeled *E. faecalis* and *L. gasseri* with ratio of 1:9, the fluorescent intensity pattern was significantly shifted, compared to the control sample treated with *E. faecalis* alone (Figure 2C).

### Probiotic bacteria decrease binding of *E. faecalis in vivo*

**Figure 3:**
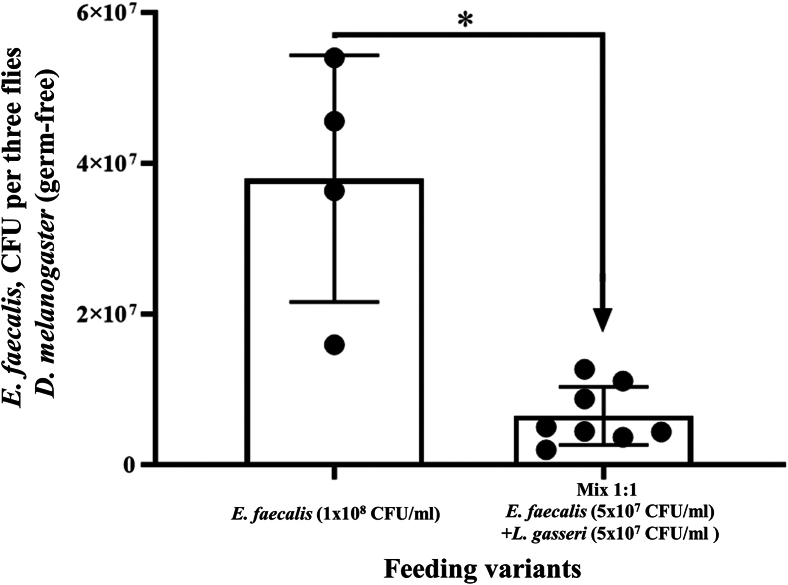
Quantification of colony-forming units (CFU) of *E. faecalis* in axenic flies following different feeding conditions “*E. faecalis*” represents the positive control, where flies were fed with *E. faecalis* alone. “Mix 1:1 *E faecalis* + *L. gasseri*” indicates feeding flies with the mixture of *L. gasseri* and *E. faecalis* in a 1:1 ratio. The results are presented as the mean ± SD. Statistical analysis of data was performed using an unpaired t-test with Welch’s correction, where ∗ (*p* ≤ 0.05) indicates a significant difference.

To examine whether mixing *L. gasseri* with *E. faecalis* can decrease the ability of *E. faecalis* to bind to the host cells, two variants of *in vivo* experiments were performed, using axenic *Drosophila melanogaster* flies and flies with native microbiota. Quantitative inoculation of sacrificed flies showed that there was a significant reduction of *E. faecalis* number per fly when flies were fed with the 1:1 mixture of opportunistic *E. faecalis* and probiotic *L. gasseri* in comparison with flies fed by the *E. faecalis* mono suspension (Figure 3).

**Figure 4:**
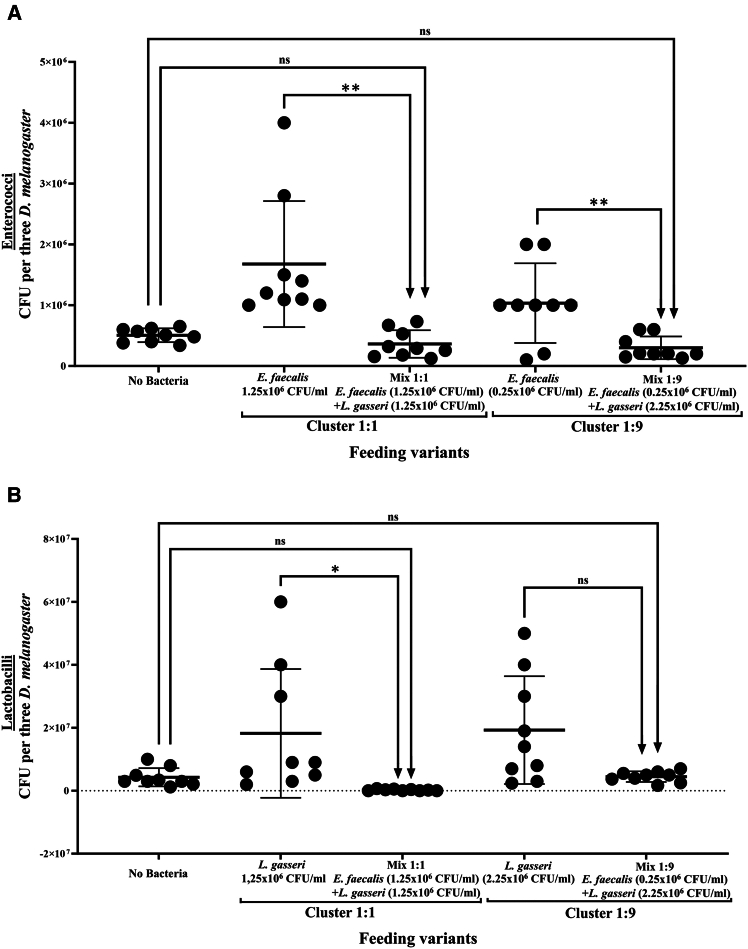
Quantification of *Enterococci* and *Lactobacilli* in *D. melanogaster* after bacterial feeding Bacterial load of enterococci (A) and lactobacilli (B) in *D. melanogaster* with native microbiota estimated after feeding with sterile sucrose solution (“no Bacteria” group), *L. gasseri* or *E. faecalis* mono suspensions, or *L. gasseri* and *E. faecalis* mixed in different proportions (“Cluster 1:1” and “Cluster 1:9” indicate the 1:1 and 1:9 ratios). The bacterial load of groups was compared using t-tests with Welch’s correction. ∗ (*p* ≤ 0.05) and ∗∗ (*p* ≤ 0.01) indicates a significant difference, while ‘ns’ denotes a non-significant result. The results are presented as the mean ± SD.

Experiment with *D. melanogaster* flies with native microbiota (including *Lactobacilli* and *Enterococci*). revealed that the level of enterococci bacterial load in the group fed with the *E. faecalis* only (both with 1.25 × 10^6^ CFU/mL and 0.25 × 10^6^ CFU/mL), was significantly higher than in the control group (“no bacteria”), with indigenous enterococci as the part of native microbiota. However, when *D. melanogaster* were fed with the same concentrations of *E. faecalis*, mixed with *L. gasseri* in a 1:1 or 1:9 ratio, the enterococci bacterial load decreased significantly compared to the corresponding groups of flies fed only with different *E. faecalis* concentrations. Although the decrease in enterococci bacterial load for both clusters was not statistically significant when compared to the control group (“no Bacteria”), fed only with a sterile 5% sucrose solution (Figure 4A).

On the contrary, the bacterial load of lactobacilli in the groups fed with *L. gasseri* 2.25 × 10^6^ CFU/mL and 1.25 × 10^6^ CFU/mL was not affected when mixed with *E. faecalis* in a 1:1 or 1:9 ratio. Notably, the load of lactobacilli in the group fed with a 1:1 mixture ratio with *E. faecalis* was significantly reduced, comparing to the native *Lactobacillus* load in the control group of flies (Figure 4B).

*Enterococci* and *Lactobacilli* were differentiated according to the appearance on the MRSA plates confirmed by the dark field microscopy (Figure S2).

To confirm the reduction in *E. faecalis* binding to the intestinal tract of *D. melanogaster* flies when combined with *L. gasseri* bacterial suspension, we conducted immunofluorescence staining on formalin-fixed paraffin-embedded (FFPE) sections of the flies with native microbiota.

**Figure 5:**
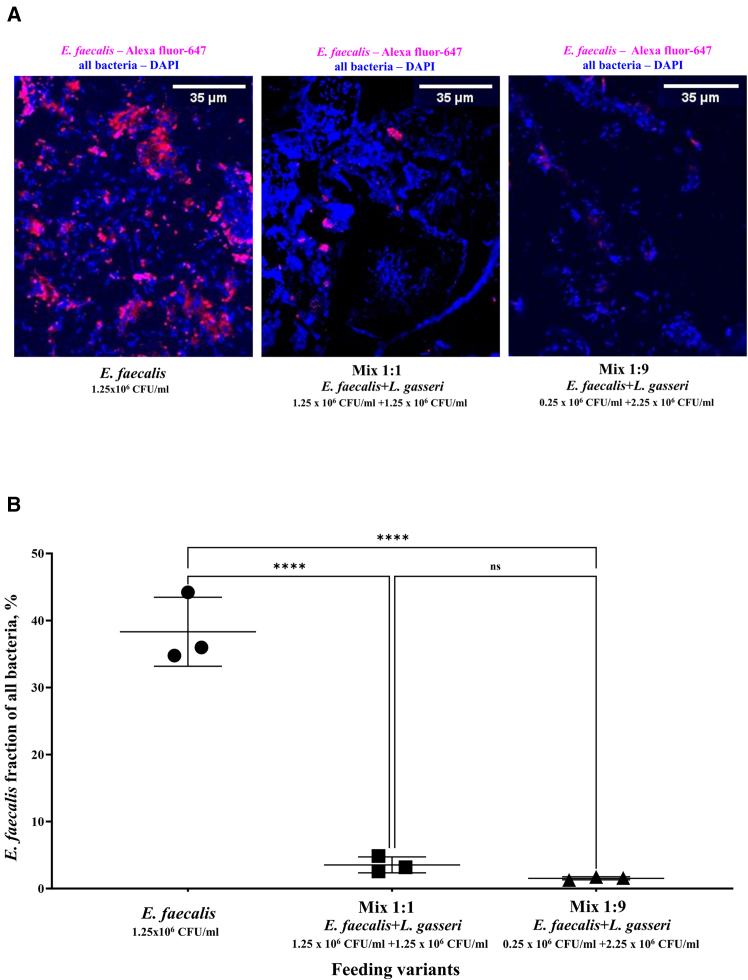
Results of IF-staining of FFPE sections of *D. melanogaster* with native microbiota after feeding with different variants of bacterial suspensions (A) Representative images of IF-stained sections of flies; a scale bar of 35 μm is provided and applies to all images in (A). (B) Counted *E. faecalis* fraction of all bacteria detected by IF-microscopy with anti-*E. faecalis* antibodies in three groups of flies which were fed by *E. faecalis* (1.25 × 10^6^/mL) only, or by a mixed bacterial suspension at a ratio of 1:1 (*E. faecalis* 1.25 × 10^6^/mL)/*L. gasseri* (1.25 × 10^6^/mL), or 1:9 (*E. faecalis* 0.25 × 10^6^/mL/*L. gasseri* 2.25 × 10^6^/mL). ∗∗∗∗ (*p* ≤ 0.0001) indicates a significant difference, while ‘ns’ denotes a non-significant result. The results are presented as the mean ± SD. Data were analyzed with Tukey’s multiple comparisons test.

Observing immunofluorescence staining microscopy images (Figure 5A) and analyzing the data statistically (Figure 5B), we found a significant decrease in *E. faecalis* levels in flies fed with a 1:1 mixture, compared to the *E. faecalis* control. Interestingly, mixing *E. faecalis* with an equal amount of *L. gasseri* resulted in the decrease that was not statistically different from the 1:9 mixture.

### N-acetylcysteine reduces the aggregation between *E. faecalis* and *L. gasseri*

It is known that N-acetylcysteine (NAC) can destroy disulphide bonds of glycoproteins that are essential for bacterial co-aggregation and biofilm formation.^57^ To reveal the possible nature of co-aggregation between *L. gasseri* and *E. faecalis*, bacterial mixtures of these two species were treated with different concentrations of NAC. FITC-labeled *E. faecalis* alone or mixed with *L. gasseri* were treated with two concentrations of NAC in MEM-α medium: 0.5 mg/mL or 5 mg/mL.

**Figure 6:**
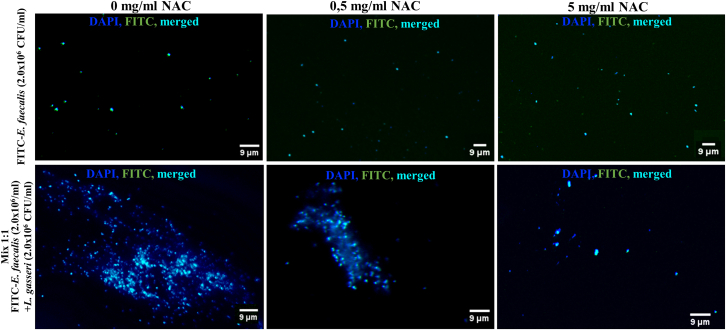
NAC disrupt aggregates of *E. faecalis* and *L. gasseri* at two different concentrations Representative images of bacterial suspensions of FITC-labeled *E. faecalis, or* FITC-labeled *E. faecalis* and *L. gasseri* incubated at designated conditions. *L. gasseri* and *E. faecalis* DNA stained by DAPI. Pictures were taken with an FCM. A scale bar of 9 μm is provided and applies to all images in this figure.

Fluorescent microscopy revealed that mono-suspensions of FITC-labeled *E. faecalis* were not able to form the aggregates. However, when FITC-labeled *E. faecalis* was mixed with probiotic bacteria, the formation of aggregates was observed. The presence of NAC inhibits the co-aggregation of bacteria in a dose-specific manner, showing that 5 mg/mL of NAC concentration halts the development of bacterial co-aggregates (Figure 6).

## Discussion

Traumatic dental injuries (TDI) pose a significant risk of pulp inflammation and consequently the development of pulp necrosis and periapical bone inflammation. This arises from the exposure of dental tissues to the oral microbiota, which under normal circumstances cannot penetrate a healthy tooth. The state of the oral microbiota, particularly in the context of oral dysbiosis and imbalance in bacterial composition can potentially escalate inflammation within the injured tooth, exacerbating the damage caused by the traumatic incident.^5^ Oral bacteria that infiltrate the root canal following trauma can hinder successful regenerative treatment, which aims to restore lost pulp tissue and facilitate proper root maturation using the patient’s dental stem cells. Therefore, maintaining and restoring the balance of the oral microbiota may be a crucial strategy in preventing opportunistic bacterial colonization of traumatized tooth tissues. In this context, in our study, we assessed aggregative properties of probiotic species with opportunistic bacteria. Our findings revealed that probiotic *L. gasseri* not only exhibits a propensity to aggregate with opportunistic bacteria but also demonstrates a capacity to impede the adhesion of opportunistic *E. faecalis* to SCAPs. Additionally, we showed that it effectively reduces the bacterial load of *E. faecalis* in an *in vivo* model of the *D. melanogaster* gut.

In the oral cavity, the phenomenon of bacterial co-aggregation, involving the adhesion of different bacterial species to each other, is a fundamental process in the formation of bacterial biofilms. This process contributes to shaping the microbiotic composition of the oral cavity.^58^ Respectively, occurrence of co-aggregation may vary between different species, according to the different nature of their co-aggregation.^59^ Specifically, the phenomenon of co-aggregation was first observed in human dental plaque bacteria in 1970s.^58^ In our study, to evaluate the co-aggregation abilities of probiotics toward oral bacteria, we utilized bacterial species that are implicated in endodontic treatment failure—*E. faecalis* in a different combination with probiotic species —*L. gasseri*. The utilized *L. gasseri* strain was isolated from breastfed infants and had previously demonstrated inhibitory activity toward other oral opportunistic bacteria.^55^ Our results showed that chosen *E. faecalis* strain poorly forms auto-aggregates, but partially co-aggregates with probiotic *L. gasseri* in different concentrations. Consistent with our study, it was shown that *E. faecalis* showed the highest co-aggregation with different *Lactobacillaceae* species and lower percentage of auto-aggregation, among other enteropathogenic bacteria.^48^

In case of traumatic incident or during endodontic treatment, stem cells from the apical papilla (SCAP) can come in contact with oral bacteria, and as was shown in our previous studies, this cell–bacteria interaction lead to the release of pro-inflammatory markers and changes in SCAPs differentiation potential toward mineralization.^60^^,^^61^^,^^62^ We have previously demonstrated that *E. faecalis* exhibit strong binding to SCAP. In contrast, probiotic species did not demonstrate an equivalent binding ability to these cells.^63^ To replicate the *in vivo* scenario where SCAP within the root canal are exposed to bacteria during TDI, our goal was to assess whether the adhesion of opportunistic bacteria can be reduced or mitigated through the application of probiotic species. As the result, we observed with flow cytometry assay that the addition of probiotic *L. gasseri* to the *E. faecalis* suspension resulted in the reduction of adhesion of *E. faecalis* to SCAP. Particularly, it was observed when the number of probiotics exceeded the number of opportunistic bacteria by a factor of nine. In contrast to our results, when examining colon cancer cell lines, it was observed that *L. gasseri’s* influence did not result in decrease of adhesion of *E. faecalis* to cells. However, other species—*L. rhamnosus*, *L. acidophilus*, *L. plantarum*, and *L. ultunensis*—all exhibited a statistically significant decrease in the binding of *E. faecalis* to the colon cancer cell lines.^48^

Exploring the *in vivo* dynamics of bacterial adhesion provides deeper insights into the intricate processes occurring within living organisms under various variables. Therefore, we utilized *D. melanogaster in vivo* model, to show the efficacy of probiotics in prevention of *E. faecalis* binding in the intestine. The utilization of the *D. melanogaster* model is considered a promising approach for studying host–microbiota interactions, owing to its affordability and convenience as an *in vivo* model.^64^ It has been previously showed that oral feeding model of *D. melanogaster* is a valuable tool to study interaction between oral bacteria.^65^ In particular, this approach was previously utilized by Bronnec and Alexeyev, in visualizing *Propionibacterium* (*Cutibacterium*) *spp*. biofilm in a *D. melanogaster* gut.^66^ Accordingly, in our study, we utilized axenic flies, as well as flies with native microbiota. Our results demonstrated that in both germ-free flies and flies with native microbiota (which already contain *Enterococci* and *Lactobacilli*^67^) *E. faecalis* load was significantly decreased when *L. gasseri* was added to the feeding mixture. To the best of our knowledge, there are no literature showing the effect of probiotics on adhesion of pathogenic bacteria *in vivo* which are based on co-aggregation phenomenon; therefore, more studies are needed. Nonetheless, it has been demonstrated that pre-feeding flies with probiotic *L. plantarum* protects them from enteropathogenic *Serratia marcescens*, thereby increasing the survival rate of flies.^68^

In our study, we implemented a robust validation of the results obtained through the traditional CFU counting method and by employing immunofluorescence staining on sections of flies’ intestines. The latter approach allowed us to visually assess the binding of *E. faecalis* to the intestinal tissues. Our findings not only corroborate the quantitative data from CFU counting but also offer a different view on the interactions at the microbial–host interface.

It is known that co-aggregation between bacteria usually imply the interaction between adhesins—lectin-like proteins on the bacteria cell surface, and polysaccharide-containing receptors located on other bacteria.^69^^,^^70^^,^^71^ Moreover, the co-aggregation between bacteria can be mediated by protein-protein interaction.^72^^,^^73^ Therefore, in our work we also aimed to hypothesize about the nature of co-aggregation that occurs between *E. faecalis* and *L. gasseri*. In this context, we examined NAC substance, abbreviated as NAC, due to its capacity to break down disulphide bonds within proteins, thereby inducing structural alterations and interrupting their ligand bonding.^74^ NAC, an FDA-approved medication, is primarily prescribed for its anti-mucus activity. Additionally, it has demonstrated efficacy as an agent against biofilm formation.^75^^,^^76^^,^^77^ With fluorescence microscopy we demonstrated that co-aggregation between probiotic *L. gasseri* and opportunistic *E. faecalis* was disrupted under the influence of NAC, revealing a correlation: as the concentration of NAC increased, the formation of co-aggregates was reduced. Previously, it was shown that the combination of NAC with levofloxacin demonstrated significant effectiveness against *E. faecalis* biofilm in the context of regenerative endodontics.^78^ *Enterococci* are known for their tendency to form biofilms, facilitated by a range of adhesins. Among these, proteins like PrgB –adhesin of *E. faecalis*– play a significant role in bacterial aggregation and virulence.^79^^,^^80^ However, since co-aggregation can occur through both protein–protein interactions and interactions between protein adhesins and polysaccharide receptors,^81^ further studies are required to precisely understand the mechanisms mediating co-aggregation between *E. faecalis* and *L. gasseri*.

Our results open a perspective of utilizing probiotic bacteria as preventive treatment in case of the occurrence of TDI or during RET, due to the effect of probiotic bacteria on bacteria that are associated with endodontic infection.^82^ Existing results are already showing the effectiveness of probiotics administration toward conditions like halitosis,^83^ caries,^84^ and complementary therapy for periodontitis.^85^ As well, implementing probiotics for improving oral health can be used in form of lozenges,^86^ mouthwashes,^87^ and intracanal irrigation.^88^ The study conducted by Gaeta et al. highlighted that *E. faecalis* in saliva as a risk factor for endodontic infection.^89^ In this context, one potential preventive measure could involve reducing the presence of *E. faecalis* in saliva through administration of probiotic for example with the *L. gasseri* strain that was used in this study. Multiple studies have shown that probiotic strains of *Lactobacillus* species are effective not only against planktonic *E. faecalis*,^90^ but also against its biofilms.^91^^,^^92^

Taken together, our findings highlight the promising clinical potential of probiotics, particularly *L. gasseri* which, due to its co-aggregative nature with oral opportunistic bacteria such as *E. faecalis*, exhibits anti-adhesive properties. This trait enables *L. gasseri* to effectively inhibit the binding of opportunistic oral bacteria to SCAP, while also demonstrating its adhesion preventive properties in a *D. melanogaster in vivo* model. Through this study, we underscore the practical application potential of probiotics, which can act on oral dysbiosis, consequently improving the outcome of RET in infected teeth.

### Limitations of the study

While acknowledging the limitations that are present in our study, such as the utilization of the *D. melanogaster* intestine as an *in vivo* model, it is crucial to recognize the distinct differences between the human oral cavity environment and the environment of the *D. melanogaster* gut. Acknowledging the differences in bacterial binding between human dentin and fly intestines, this model has been used to study the interactions between multiple oral bacterial species. In these models, fruit fly intestines served as a reservoir for bacterial colonization to study oral bacteria biofilm formation abilities.^65^^,^^93^^,^^94^^,^^95^ Nevertheless, our study successfully elucidated the co-aggregative and anti-adhesive properties of probiotic bacteria toward oral opportunistic bacteria, in both *in vitro* and *in vivo* models.

## Resource availability

### Lead contact

All data are available in the main text or the supplemental information. Further information and requests for resources and reagents should be directed to and will be fulfilled by the lead contact, Nelly Romani Vestman (nelly.romani.vestman@umu.se).

### Materials availability

This study did not generate new unique reagents.

### Data and code availability

• The bacterial 16S rRNA sequencing data of the utilized strains are publicly available and stored in the GenBank repository (https://www.ncbi.nlm.nih.gov/genbank/) with the following accession numbers: GenBank: PV404125.1 and GenBank: PV404124.1 for *E. faecalis*, GenBank: PV405038.1 and GenBank: PV405039.1 for *L. gasseri.*
• The paper does not report original code.
• Any additional information required to reanalyze the data reported in this paper is available from the lead contact upon request.

## Acknowledgments

We acknowledge the Biochemical Imaging Center at Umeå University and the National Microscopy Infrastructure, NMI (VR-RFI 2019-00217) for providing assistance in microscopy.

This research was funded by the Region of Vasterbotten (Sweden) via TUA grant number 977100 (N.V.), ALF grant number RV-967705 (N.V.), RV-996277 (M.L.) and Kempestiftelserna Kempe JCSMK23-0158.

## Author contributions

V.Z.: Methodology, writing – original draft. O.R.: Conceptualization, data curation, formal analysis, methodology, writing – original draft. A.S.: Methodology, writing – review and editing; V.B.: Methodology, writing – review and editing; N.L.: Methodology, writing – review and editing; M.B.: Funding acquisition, writing – review and editing. P.K.: Writing – review and editing. M.L.: Funding acquisition, writing – review and editing. N.R.V.: Conceptualization, funding acquisition, project administration, supervision, writing – review and editing.

## Declaration of interests

The authors declare no conflict of interests and any potential financial conflicts of interest that any of the authors may have.

## STAR★Methods

### Key resources table

**Table:** 

REAGENT or RESOURCE	SOURCE	IDENTIFIER
**Antibodies**		

Rabbit polyclonal antibodies specific for *E. faecalis*	MERCK	Cat# SAB4200868-25UL
Donkey anti-rabbit Alexa Fluor-647™ IgG	Invitrogen	RRID: AB_2536183

**Bacterial and virus strains**		

*E. faecalis* Tand-4F	Isolated from a patient^96^	GenBank: PV404125.1 GenBank: PV404124.1
*L. gasseri* B16	Isolated from a patient^55^	GenBank: PV405038.1 GenBank: PV405039.1

**Chemicals, peptides, and recombinant proteins**		

Fluorescence mounting medium	Dako, Agilent Tech	#S3023
4′,6-diamino-2-phenylindole, dihydrochloride (DAPI)	RnD Systems	Cat# 5748/10
Alexa Fluor™ 488 Phalloidin	Invitrogen	Cat# A12379
N-acetyl-cysteine (NAC)	Sigma-Aldrich	Cat# A7250-100G
Fluorescein isothiocyanate	Sigma-Aldrich	Cat# F7250-100MG

**Experimental models: Cell lines**		

Stem cells from the apical papilla (SCAP)	Isolated from patients^97^^,^^98^	N/A

**Experimental models: Organisms/strains**		

*Drosophila melanogaster* with native microbiota	Fly Collection, Odesa I.I. Mechnikov National University	Line vestigial (vg)
Axenic fruit flies generated from wild-type *Drosophila melanogaster* genotype W1118 iso; 2-iso; 3-iso; no gender distinction	Bronnec and Alexeyev,^66^ Bronnec and Alexeyev^99^	N/A

**Software and algorithms**		

ImageJ v.1.47 software	Schneider et al.^100^	https://imagej.net/ij/download.html
FlowJo software V9	Tree Star	https://www.flowjo.com/
GraphPad Prism 7.0	GraphPad Software Inc. San Diego, USA	https://www.graphpad.com/

### Experimental model and study participant details

#### Bacterial strains and culture conditions

Probiotic bacteria *Lactobacillus gasseri* B16 was used in this study. Regarding the choice of probiotic, *L. gasseri* B16 strain has been previously isolated from the oral cavity of healthy infants. *L. gasseri* B16 exhibited probiotics traits of growth inhibition of commonly found oral bacteria, as well as attaching to human epithelial cells and saliva.^55^

Opportunistic pathogen *Enterococcus faecalis* Tand-4F was isolated from the root canal of traumatised teeth of patients from the Endodontic Department, Region of Västerbotten, Sweden (Reg. no. 2016/520-31). *E. faecalis* was chosen as it is often associated with root canal treatment failure.^101^

Bacterial samples were collected, processed, characterised, and identified by comparing 16S rRNA gene sequences to databases (HOMD) as previously described.^96^

All bacterial species were stored at −80°C. *E. faecalis* were plated on fastidious anaerobic agar (FAA) plates (Svenska LABFAB, ACU-7531A) with the addition of a synthetic water-soluble vitamin K1 analogue (MERCK, M5750) to 1% concentration and 5% Sterile Defibrinated House Blood (Håtunalab, Sweden). *L. gasseri* strain was grown on MRS agar plates (Sigma Aldrich, 69964-500G). The bacterial species that were used in this study were cultured under an anaerobic atmosphere (5% CO_2_, 10% H_2_, 85% N_2_) in a in Whitley A35 Anaerobic Workstation VWR, #710-01478 at 37°C for 5–7 days.

#### Stem cells and culture conditions

In this study, SCAP were obtained from teeth of three healthy donors, (1 male and 2 females, with mean age 17 years and range 11–20 years) due to retention and lack of space.^97^^,^^98^ SCAP were cultured in Minimum Essential Medium alpha modification (MEM-α) and GlutaMAX™ (Thermo-Fisher Scientific, #32561-094) and supplemented with 10% Foetal Bovine Serum (FBS) (Sigma-Aldrich, #F7524) and 1% Antibiotic–Antimycotic Solution (Sigma-Aldrich, #P0781) in a 75cm^2^ tissue culture plastic flask (Thermo-Fisher Scientific, #83.3911.002). Cells were cultured at 37°C with atmospheric conditions of 5% CO_2_ until they reached 90% confluency, changing the culture medium every second day, SCAPs of passages 5 to 7 were used in this study. The multipotent stromal cells were authenticated by the expression of CD73, CD90, CD105, and CD146, along with the absence of CD11b, CD19, CD34, CD45, and HLA-DR. The collection, culture, storage, and use of all cell lines were approved by the local research ethics committee at Umeå University (Reg. no. 2013-276-31M). The studied SCAP was confirmed to be free of Mycoplasma species using the PCR-based Venor GeM Mycoplasma Detection Kit (Sigma-Aldrich, St. Louis, MO, USA, MP0025). For experiments utilizing SCAP, three biological replicates with two technical replicates per biological replicate were used, resulting in a sample size of six for each treatment variant. SCAP from each donor were exposed to a set of bacterial suspensions, ensuring that each experimental group included cells from a unique donor treated with the same set of bacterial suspensions.

#### Ethics considerations

All procedures were conducted in accordance with the guidelines of the ethics committee at Umeå University (Reg. no. 2013-276-31M) approved the collection, culture, storage, and usage of all cell lines, which are in compliance with the Declaration of Helsinki (64th WMA General Assembly, Fortaleza, October 2013).

#### *D. melanogaster* microbiota model

Drosophila-related experiments were conducted in Umeå University and Odesa I. I. Mechnikov University. Two lines of fruit fly *Drosophila melanogaster* were used in this study: germ-free *D. melanogaster*^66^ and *D. melanogaster* with native microbiota. Both groups of flies were maintained on a sugar-yeast medium according to Scopura et al.^102^ Before conducting experiments, flies were placed in empty tubes for one hour to stimulate appetite.^103^ For additional details, please refer to the method details section.

### Method details

#### Aggregation assay

Bacteria grown for 5 days were harvested and individual species suspensions were prepared in MEM-α cell culture medium containing 10% FBS, without antibiotics. The optical density of these suspensions was adjusted to OD_600_ = 1.0 using spectrophotometer (Ultrospec 2100 pro Amersham Biosciences). The quantification of bacteria per millilitre of suspensions with OD_600_ = 1.0 was performed through the quantitative inoculation of suspensions diluted ten-fold. To facilitate the visualisation of bacterial aggregates, 10μL of 0.4% trypan blue solution (Invitrogen, #1834791) was added per each millilitre of suspension, including the control medium. The two-fold dilution series of individual bacterial suspensions in the cell culture medium were set up in the 96-well plate (Sarstedt, #82.1582) according to the scheme of the plate (from 2 × 10^7^ to 1.56 × 10^5^ CFU/mL) to check the auto-aggregation potential of examined bacterial species. To assess the ability of *Lactobacillus* to co-aggregate with *E. faecalis*, two-fold dilutions of *L. gasseri* (ranging from 2×10^7^ to 1.56 × 10^5^ CFU/mL) were prepared. Each *E. faecalis* concentration, from 2×10^7^ to 1.56 × 10^5^ CFU/mL, was then combined with each *L. gasseri* dilution. Mixed suspensions in the wells were gently pipetted and plates were incubated at +37°C in a humid atmosphere at least 18 hours. Observations of aggregation layers or pellets formed by sedimented bacteria on the bottom of the well were made with Stereo Zoom Microscope Nikon SMZ800 microscope using Photonic PL 2000 Microscope Fibre Optic Light source. Plates were photographed with a Nikon Coolpix 4500 Camera connected to the microscope.

Evaluation of bacterial aggregation was performed by qualitative method, based on the previously known agglutination assay.^104^ Aggregation was considered positive if a uniform layer of bacteria was present at the bottom of the well. Specifically, in case of aggregation, the bacterial cell in a planktonic stage attaches to a bacterial cell of their own type (auto-aggregation), or to another bacterial species (co-aggregation) via the structures located on the bacterial cell surface. In turn, aggregation leads to the formation of biofilm, which settles to the bottom of the well in the form of a thin layer, sometimes called an ‘umbrella’. If aggregation does not occur, bacterial cells settle to the bottom of the well in the form of a small spot (sometimes called a ‘button’).^105^

#### FITC-labelling of bacteria

The labelling of bacterial species was carried out as described previously by Rakhimova et al.^63^ Derived from the cryostocks, bacterial strains were plated on FAA or MRSA, and passaged three times for 1 week, culturing in anaerobic conditions at 37°C. Afterwards, bacteria were harvested, washed by centrifugation at 7,000 × g for 10 min at 4°C, and resuspended in phosphate-buffered saline (pH 7.4) containing 0.05% Tween 20 (PBS-T). Prepared bacterial suspensions were adjusted to an optical density (600nm) of 1, via the addition of sodium carbonate buffer (pH 9.0). Bacterial inoculation was verified through quantification of colony-forming units (CFU). For labelling, 0.1mg of fluorescein isothiocyanate (FITC) (Sigma-Aldrich, #F7250-100MG) dissolved in 0.01mL di-methyl-sulfoxide (DMSO, Thermo Fisher Scientific, #D12345) was added to each millilitre of different bacterial suspensions and incubated with rotation for 10 min at room temperature. Following the incubation period, bacteria were centrifuged at 7,000 × g for 5 minutes and washed five times with PBS-T to eliminate any unbound FITC. Afterwards, labelled bacteria were reconstituted in PBS-T supplemented with 1% bovine serum albumin. The final OD of bacterial suspension was adjusted to 1.0 at 600nm. Prepared suspensions of the FITC-labelled bacteria were aliquoted and stored at −20°C.

#### *In vivo* experiment with fruit flies

##### Bacterial preparation

Bacterial strains *L. gasseri* and *E. faecalis* were grown as described above, followed by culture in brain heart infusion broth (Sigma-Aldrich, CM1135B) supplemented with 2g/L glucose (BHIg) for 48h under anaerobic conditions at 37°C. Bacteria were then harvested, resuspended in 5% sucrose solution (according to Blum et al., 2013) with the optical density of suspensions adjusted with spectrophotometer (SmartSpec, Bio Rad) to OD_600_ = 1.0 which corresponds to 1 × 10^8^ CFU/mL. Suspensions were prepared with single bacterial strain *L. gasseri* or *E. faecalis* as well as the mixture of *E. faecalis* and *L. gasseri* in different proportions: 1:1 or 1:9, respectively. Fresh suspensions were prepared daily, ensuring the viability of bacteria through growth on MRS agar plates (Sigma Aldrich, #69964-500G).

##### The germ-free flies experiment model

For the germ-free flies experiment, *D. melanogaster* genotype W1118 iso; 2-iso; 3-iso were used to generate the germ-free line. Axenic flies of both sexes were fed either with a mono-suspension of *E. faecalis* 1 × 10^8^ CFU/mL (group 1, n = 15), or mixed suspension of *E. faecalis* and *L. gasseri* (1:1) 5 × 10^7^ to 5 × 10^7^ CFU/mL (group 2, n = 20). Flies were fed bacterial suspensions only once and were sacrificed 3 days later. Euthanasia was achieved by quick freezing of the flies in vials in freezer. The sacrificed flies were superficially disinfected by submerging them in 70% ethanol for 1 minute, followed by rinsing in sterile water for 2 minutes. Subsequently, three to five flies from each group were fixed in 4% paraformaldehyde (PFA) and paraffin-embedded to be used later for the immunofluorescent experiments.

Under sterile conditions, flies were homogenised with a pestle in 100μL of sterile TE buffer. Decimal dilutions were prepared, and the samples were plated on MRS agar before culturing in anaerobic conditions (5% CO2, 10% H2, 85% N2, 37°C) for 48 hours. After culturing, colonies of *E. faecalis* and *L. gasseri* were counted, and the data were analysed using the statistical program Prism (GraphPad Software Inc. San Diego, USA).

##### Experiment model of flies with native microbiota

Similarly, wingless *D. melanogaster* flies maintained on a sugar–yeast medium were sorted by sex and divided into experimental groups consisting of 30 flies each (15 males, 15 females). Freezing anaesthesia at −20°C was used to collect the flies. Experimental groups were fed once with bacterial suspensions, while control groups were fed an equivalent volume of 5% sucrose.^68^ Following this, flies were maintained for 5 days on a sugar–yeast medium.

After 5 days, three random flies from each group (one male, two females) were frozen at −20°C, and underwent the same disinfection, grinding, plating, and culturing procedures as the germ-free flies on MRS agar. Three to five flies from each group of flies with native microbiota were fixed in 4% paraformaldehyde (PFA) and paraffin-embedded. Three independent experiments, each with three repeats, were carried out, and results were processed by GraphPad Prism 7.0 The groups of *Drosophila melanogaster* with native microbiota (including *Lactobacilli* and *Enterococci*) were fed as stated in the Table S1. Groups of *D. melanogaster* flies were fed with different bacterial suspension treatments in 5% sucrose solution.

#### Bacterial aggregation in presence of N-acetyl cysteine

Equal volumes (20μL) of *E. faecalis* (OD_600_ =0.2) suspensions were mixed with freshly prepared aliquots of *L. gasseri* (OD_600_ = 0.2) in 60μL of cell culture medium MEM-α GlutaMAX™, supplemented with 10% (FBS; GIBCO). Alternatively, a suspension containing only 20μL of FITC-labelled *E. faecalis* was prepared using 80μL of cell culture medium.

The suspensions were incubated for 60 minutes without the addition of N-acetyl-cysteine (NAC, Cat. no. A7250-100G, Sigma-Aldrich) or with one of two different NAC concentrations: 0.5mg/mL or 5.0mg/mL, for 60 minutes at +4°C on a rocking platform. It is important to note that the ability of NAC in these concentrations to lower the pH of the cell culture medium was pretested due to potential bleaching of FITC fluorescence at pH values below 5.0.

Subsequently, DAPI (RnD Systems, #5748/10) was added to all variants of suspensions to stain bacterial cell nuclei. Treatment and bacterial suspensions from two replicate tubes were applied to microscopy slides and analyzed immediately with a fluorescence microscope (Olympus BX41TF Fluorescence Microscope) for blue and green fluorescence simultaneously as described above. Ten fields of view were investigated for each variant of treatment. At least three images for each variant of treatment were taken.

### Quantification and statistical analysis

#### Flow cytometry of SCAP mixed with bacterial suspensions

SCAP from passages 4 to 6 were harvested through trypsinisation in the presence of EDTA (Sigma-Aldrich, #T3924) when confluency reached 80%–90%. Harvested cells were counted by automated cell counter (Countess™ Automated Cell Counter, Thermo Fisher Scientific) and resuspended in MEM-α to 1 × 10^6^ cells/mL. SCAPs were exposed to *E. faecalis* alone, and in different proportions with probiotic bacteria. For each bacterial strain, 0.1mL of FITC labelled bacteria (1 × 10^8^ CFU mL) was applied to 0.1mL of cell suspension (1 × 10^6^/mL) to reach a multiplicity of infection (MOI) of 1:100 followed by incubation in darkness on a rocking table for 2h at 4°C. After incubation, cells were washed three times by centrifugation at 400 × g for 5 minutes and resuspension in PBS. Thereafter, SCAP with bound bacteria were resuspended in 500mL of PBS, and kept at 4°C until analysed by flow cytometry (FC) within 1h. FC analysis of SCAPs with or without bound bacteria was carried out using a Becton-Dickinson Accuri™ C6 device (BD Biosciences), equipped with a 488nm blue laser. The forward and side scatters were utilised to create a cell population gate, excluding the FITC signal originating from unbound bacteria. The fluorescence signal (FL1-A; 533/30 nm) was measured to calculate bound bacteria.

Fluorescence values were obtained from the acquisition of 10,000 gated cells. FlowJo software V9 (Tree Star) was used to analyse the data. Representative results are shown from at least three independent experiments.

#### Visualisation of *E. faecalis* and *L. gasseri* aggregates in the gastric tract of *D. melanogaster*

After three days feeding flies with different bacterial suspensions, cold anaesthesia was used to immobilise the fruit flies, followed by fixation in formalin and subsequent paraffin embedding. The paraffin blocks were sliced into 4μm sections and affixed to SuperFrost Plus GOLD white adhesion slides (Fisher Scientific). Sections were deparaffinised in xylene, rehydrated in ethanol series (100%, 70%, 30%), and used for immunofluorescent microscopy. To detect *E. faecalis* that were bound to gastric tract of flies, fruit fly sections were incubated with primary antibody—anti-*E. faecalis* rabbit polyclonal antibodies (Merck SAB4200868-25UL). Incubation with primary antibody were followed by incubation with fluorescently labelled secondary conjugates—donkey anti-rabbit Alexa Fluor-647 IgG (Invitrogen A31573), used in a 1:100 dilution. Alexa Fluor™ 488 Phalloidin by Invitrogen was used to stain and visualise actin filaments of *D. melanogaster.* All flies section samples were labelled with 4′,6-diamino-2-phenylindole, dihydrochloride—DAPI (RnD Systems, #5748/10) in a 1:3500 dilution. The sections were analysed on a Zeiss Axio Imager M2 microscope (Carl Zeiss Vision).

#### Statistical analysis

The experiments were performed with three biological replicates and two technical replicas in each variant of treatment to reduce experimental error. The results are expressed as a mean ± SD. For analysis of IF-microscopy data, Tukey’s multiple comparison test was used. FC results were processed with Dunnett’s multiple comparisons test. Statistically significant results are marked with an asterisk (∗), where ∗ (*p* < 0.05), ∗∗ (*p* < 0.001), or ∗∗∗∗ (*p* < 0.00001). GraphPad Prism 7.0 (GraphPad Software Inc. San Diego, USA) software package were used for the statistical analysis.
