# Supplementary material for: Inhibition of infection-associated oral bacteria adhesion by probiotics: In vitro and in vivo models
Source: iScience. 2025 Apr 11;28(5):112412. doi: 10.1016/j.isci.2025.112412 (PMC12245439; doi:10.1016/j.isci.2025.112412)
Supplement: Document S1. Figures S1 and S2 and Table S1 [file mmc1.pdf]

iScience, Volume *28*

Supplemental information

Inhibition of infection-associated oral bacteria adhesion by probiotics: *In vitro* and *in vivo* models

Valeriia Zymovets, Olena Rakhimova, Alexej Schmidt, Vicky Bronnec, Nataliia Limanska, Malin Brundin, Peyman Kelk, Maréne Landström, and Nelly Romani Vestman

Begränsad delning

Supplementary material

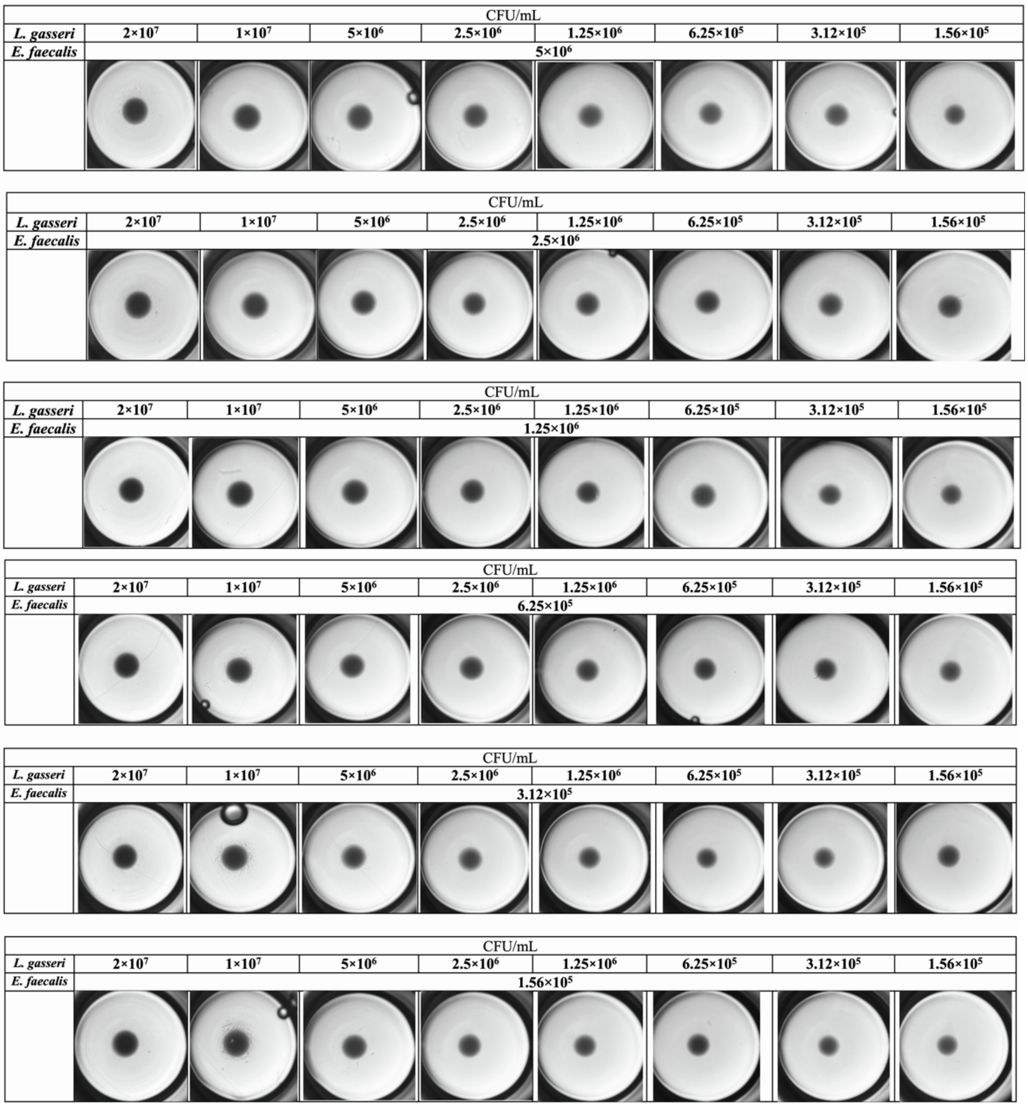

Figure S1. Aggregation test of *L. gasseri* at gradual two-fold dilutions in combination with different concentrations of *E. faecalis*.

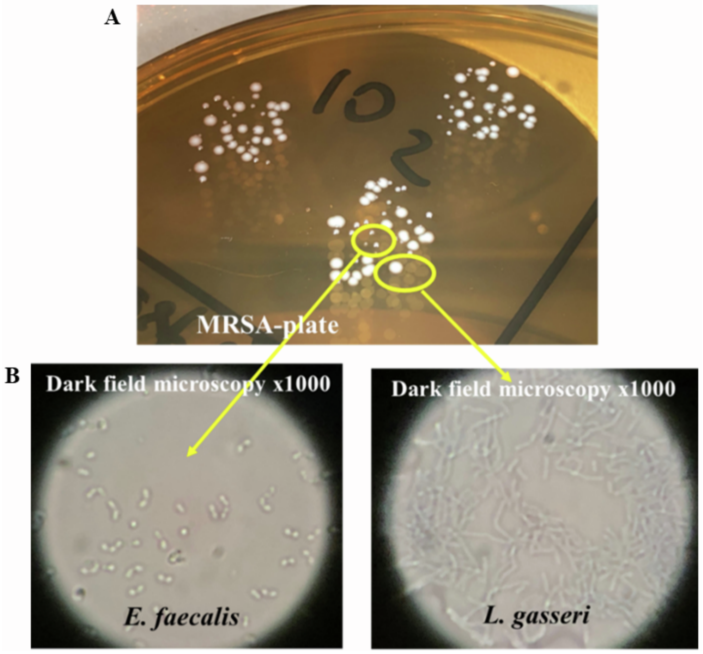

Figure S2. Cultured colonies of *E. faecalis* and *L. gasseri* from sacrificed flies **(A)** on MRSA plates and **(B)** dark-field microscopy.

Table S1. Groups of *D. melanogaster* flies fed with different bacterial suspension treatment variants.

	Cluster 1:1	Cluster 1:9
**Gr. 1 "No bacteria" (n=30): 5 % sucrose solution;**	**Gr. 2** "*E. faecalis* 1.25 x 10^6 CFU/mL" (n=30): high mono suspension of *E. faecalis*;	**Gr. 5** "*E. faecalis* 0.25 x 10^6 CFU/mL" (n =30): low mono suspension of *E. faecalis*;
	**Gr. 3** "*L. gasseri* 1.25 x 10^6 CFU/mL" (n=30): high mono suspension of *L. gasseri*;<br><br>**Gr. 4** "Mix 1:1 *E. faecalis* (1.25x10^6 CFU/mL)+*L. gasseri* (1.25 x 10^6 CFU/mL) " (n =30): mixtures of *E. faecalis* and *L. gasseri* in a ratio 1:1 ;	**Gr. 6** "*L. gasseri* 2.25 x 10^6 CFU/mL" (n =30): high mono suspension of *L. gasseri*;<br><br>**Gr. 7** "Mix 1:9 *E. faecalis* (0.25 x 10^6 CFU/ml)+ *L. gasseri* (2.25 x 10^6 CFU/mL) " (n =30): mixtures of *E. faecalis* and *L. gasseri* in a ratio 1:9;
